# Increased pin diameter improves torsional stability in supracondylar humerus fractures: an experimental study

**DOI:** 10.1007/s11832-016-0722-z

**Published:** 2016-03-14

**Authors:** Anupam Pradhan, William Hennrikus, Gregory Pace, April Armstrong, Gregory Lewis

**Affiliations:** Pennsylvania State College of Medicine, 30 Hope Drive, Hershey, PA 17033 USA

**Keywords:** Supracondylar, Elbow fracture, Pediatric, Humerus, Pin diameter

## Abstract

**Background:**

Pediatric supracondylar humerus fractures are the most common elbow fractures seen in children, and account for 16 % of all pediatric fractures. Closed reduction and percutaneous pin fixation is the current treatment technique of choice for displaced supracondylar fractures of the distal humerus in children. The purpose of this study was to determine whether pin diameter affects the torsional strength of supracondylar humerus fractures treated by closed reduction and pin fixation.

**Methods:**

Pediatric sawbone humeri simulating a Gartland type III fracture were utilized. Four different pin configurations were compared. Specimens were subjected to a torsional load producing internal rotation of the distal fragment. The stability provided by 1.25- and 1.6-mm pins was compared.

**Results:**

The amount of torque required to produce 15° and 25° of rotation was greater using larger diameter pins in all models tested. The two lateral and one medial large pin (1.6 mm) configuration required the highest amount of torque to produce both 15° and 25° of rotation.

**Conclusions:**

In a synthetic pediatric humerus model of supracondylar humerus fractures, larger diameter pins (1.6 mm) provided increased stability compared with small diameter pins (1.25 mm). Fixation using larger diameter pins created a stronger construct and improved the strength of fixation.

## Introduction

Pediatric supracondylar humerus fractures (SCH) are the most common elbow fractures seen in children [1]. Displaced SCH Gartland types II and III are typically treated with closed reduction and percutaneous pin fixation [2–10]. Despite advances in treatment, loss of fixation still occurs in up to 6 % of cases, likely due to poor quality of reduction and poor fixation, resulting in malunion and limb deformity [11–13]. Previous biomechanical studies have focused on the most stable pin construct and on the number of pins necessary when treating a displaced supracondylar fracture of the distal humerus, showing that inclusion of a medial pin contributes greatly to the overall strength of fixation [14–17]. Previous biomechanical studies have demonstrated that the maximum stability for fracture fixation is provided by crossed pins placed from the medial and lateral condyles [14–17]. However, few studies have assessed the effect of pin diameter on the torsional strength of the treated fracture [18–20]. The purpose of our study was to determine whether pin diameter affects the torsional strength of supracondylar fractures treated by closed reduction and percutaneous pinning.

## Materials and methods

Each sawbone humerus (Sawbones #1052, pediatric humerus 26 cm, Pacific Research, Vashon Is., WA, USA) was osteotomized transversely at the mid-olecranon fossa with a 2-mm oscillating saw to simulate a Gartland type III fracture. The osteotomy was then reduced and stabilized with pins using a hand-held power wire driver. Four pin configurations were compared: two lateral, three lateral, one lateral and one medial, and two lateral and one medial pins. We compared 1.25- and 1.6-mm smooth stainless steel pins for each group (Synthes, Paoili, PA, USA). A total of eight test groups was therefore included. Ten humeri were tested in each configuration, making a total of 80 humeri. Bi-cortical fixation of both fragments was achieved with each pin, and lateral pins were placed in divergent fashion.

To test each construct, the Interlaken/MTS axial torsion machine (MTS: Eden Prarie, MN, USA) was utilized. The fixed specimens were subjected to a torsional load producing internal rotation of the distal fragment. Rotation in degrees and the corresponding torque were measured. We applied a torsional rotation of 1°/s for >30° of rotation, and torque measurements were recorded (in newton-meters) at a frequency of 20 Hz.

Internal rotation was selected because this direction of rotation reproduces a common clinical direction of rotation that can lead to failure. Construct failure was defined as disruption of the pin/sawbone interface. Continuous variables were reported as means with standard deviations (SD). Group comparisons were made using the unpaired Student’s *t* test. Two-sided *p* values were considered statistically significant when <0.05.

## Results

The torque (N m) required to produce 15° of rotation and 25° degrees of rotation was greater using larger diameter pins. These results are charted in Table 1.

**Table 1:** Torque

Pin configuration	No. of specimens	Torque (N m)	
		15° Rot	25° Rot
2 lateral (s)	10	0.5 ± 0.05	0.7 ± 0.06
2 lateral (l)	10	0.6 ± 0.08	0.9 ± 0.13
3 lateral (s)	10	0.8 ± 0.10	1.1 ± 0.15
3 lateral (l)	10	0.9 ± 0.11	1.4 ± 0.16
1 medial (s) and 1 lateral (s)	10	1.1 ± 0.07	1.6 ± 0.13
1 medial (l) and 1 lateral (l)	10	1.5 ± 0.11	2.2 ± 0.15
2 lateral (s) and 1 medial (s)	10	1.3 ± 0.08	2.0 ± 0.08
2 lateral (l) and 1 medial (l)	10	1.6 ± 0.09	2.5 ± 0.14

Values are given as means and standard deviations

*s* Small, *l* large, *Rot*. rotation

In the two lateral pin model, the torque required to produce 15° of rotation was 0.50 N m for the small pins and 0.64 N m for the large pins (*p* = 0.08). Similarly, in the two lateral pin model at 25°, torque for the small pins was 0.68 N m compared to 0.91 N m for the large pins (*p* = 0.07). In the three lateral pin model at 15°, torque for the small pins was 0.78 and 0.87 N m for the large pins (*p* = 0.33). In the three lateral pin model at 25°, torque for the small pins was 1.14 and 1.35 N m for the large pins (*p* = 0.11). In the one lateral and one medial pin model at 15°, torque for the small pins was 1.14 and 1.47 N m for the large pins (*p* = 0.01). In the one lateral pin and one medial pin model at 25°, torque for the small pins was 1.58 and 2.23 N m for the large pins (*p* = 0.002). In the two lateral and one medial pin model at 15°, torque for the small pins was 1.29 and 1.60 N m for the large pins (*p* = 0.02). Finally, in the two lateral and one medial pin model at 25°, torque for the small pins was 2.04 and 2.50 N m for the large pins (*p* = 0.02).

In comparing the small pin models to the matched large pin models, the differences were found to be statistically significant at 15° (Table 2) and 25° (Table 3) in both the one lateral and one medial pin model as well as the two lateral and one medial pin model. Furthermore, there was a trend towards greater stability using larger pins in comparison to smaller pins in the two lateral and three lateral pin models at both 15° and 25° of rotation. These trends, although strong, did not reach statistical significance.

**Table 2:** Mean difference in torque between small and large pins at 15°

Pin configuration	Mean (SD)	Pin configuration	Mean (SD)	*p* value
2 lateral (s)	0.5 ± 0.04	2 lateral (l)	0.6 ± 0.08	0.08
3 lateral (s)	0.8 ± 0.1	3 lateral (l)	0.9 ± 0.11	0.33
1 lateral (s) and 1 medial (s)	1.1 ± 0.07	1 lateral (l) and 1 medial (l)	1.5 ± 0.11	0.01
2 lateral (s) and 1 medial (s)	1.3 ± 0.08	2 lateral (l) and 1 medial (l)	1.6 ± 0.09	0.02

Mean torque values are in newton-meters (N m)

*SD* standard deviation, *s* Small, *l* large

**Table 3:** Mean difference in torque between small and large pins at 25°

Pin configuration	Mean (SD)	Pin configuration	Mean (SD)	*p* value
2 lateral (s)	0.7 ± 0.06	2 lateral (l)	0.9 ± 0.13	0.07
3 lateral (s)	1.1 ± 0.15	3 lateral (l)	1.4 ± 0.16	0.11
1 lateral (s) and 1 medial (s)	1.6 ± 0.13	1 lateral (l) and 1 medial (l)	2.2 ± 0.15	0.002
2 lateral (s) and 1 medial (s)	2.0 ± 0.08	2 lateral (l) and 1 medial (l)	2.5± 0.14	0.02

Mean torque values are in newton-meters (N m)

*SD* standard deviation, *s* Small, *l* large

When examining stability with regards to pin construct, the study also demonstrated that at both 15° and 25° of rotation, the configurations including a medial pin were more stable than those without (*p* < 0.001). In these samples, two lateral and one medial pin was the most stable construct overall, followed by one lateral and one medial pin, three lateral pins, and lastly two lateral pins. This finding was true whether comparing large pin models or small pin models.

## Discussion

Supracondylar fractures of the humerus represent 50–70 % of all elbow fractures in children in the first decade of life [21]. The standard of care for Gartland type II and III SCH fractures involves closed reduction with percutaneous pinning [22]. Recent biomechanical studies have emphasized the advantage of crossed-pin fixation even with the increased risk of iatrogenic ulnar nerve injury from the medial pin [16, 17, 19]. However, few studies have assessed the effect of pin diameter on the torsional strength of the treated fracture [20]. The results from our current study indicate that larger pin size results in increased stability of SCH fractures.

The results of our current study are in agreement with other studies assessing the effect of pin size on fracture stability. A biomechanical study by Srikumaran et al. comparing the stability of 1.6- and 2.8-mm pins in various configurations concluded that large pins in any configuration provided more stable reduction in sagittal extension bending than small pins [19]. However, our study differs from theirs in several important ways. First, the use of 2.8-mm pins is not clinically relevant, as pins larger than 1.6 or 2.0 mm are rarely used for this type of fracture fixation. Second, we specifically chose to use a model of torsional resistance to rotational displacement similar to the methods described by Zionts et al. in their landmark paper on the torsional strength of pin configurations [14]. According to Zionts et al., torsional loading simulates the loading that occurs clinically when the portion of the arm distal to the SCH fracture is internally rotated. Similarly, a biomechanical study by Gottschalk et al. comparing 1.6- and 2.0-mm pins found that a larger pin construct provides improved resistance to rotational stress; however, they only assessed lateral pin configurations [20]. Finally, in a separate study, Srikumaran et al. retrospectively reviewed the outcomes of pediatric patients treated for Gartland type III SCH fractures and found that patients treated with large pins were more likely to maintain sagittal alignment at final follow-up [18].

It is well known from previous biomechanical studies that the addition of a medial pin providing crossed-pin fixation improves the stability of SCH fractures, and that the use of two lateral pins alone is associated with a higher likelihood of loss of fixation [6, 12, 14]. In line with these findings, our results showed that the two crossed-pin configurations were stronger than the configurations using lateral pins alone. Furthermore, when comparing the amount of torque required to produce 15° and 25° of rotation, the crossed-pin configurations using small pins were stronger than both lateral pin configurations using large pins. Although not reaching significance, there was a trend of increasing stability of the lateral pin configurations with increasing pin size. This would suggest that the use of larger (>1.6 mm) laterally placed pins may reduce the need for a medial pin, which risks injury to the ulnar nerve, and therefore lateral-only pinning improves safety [23, 24]. According to a systematic review by Slobogean et al., there is an iatrogenic ulnar nerve injury for every 28 patients treated with crossed pinning compared with lateral pinning [25]. Current American Academy of Orthopaedic Surgeons guideline recommendations for the treatment of pediatric SCH fractures are for two or three lateral pins with avoidance of the medial pin [26]. From a clinical standpoint, the surgeon is focused on obtaining a good reduction and stable pin fixation. In most cases in the clinical situation a medial pin is not needed unless the fracture remains unstable following lateral-only pinning.

This study has a few limitations. For one, the use of sawbone models does not take into account the surrounding anatomical structures, such as the periosteum, that may contribute to fracture stability, nor does it accurately reflect the variable presentation of supracondylar humerus fractures. Furthermore, the sawbone is not representative of pediatric bone. It is also important to note that in the clinical situation all patients are given a supplemental cast or splint following fixation, which adds to the mechanical stability of such a construct and more importantly prevents exertion of mechanical forces including rotational torque or axial distraction following fixation. Additionally, the mechanism of stress applied in our study does not necessarily accurately reflect all of the physiologic stresses the elbow experiences during healing. The main benefit of using synthetic models is their uniform nature, thereby allowing for isolation of the variables being tested: in our case the pin size and configuration.

In conclusion, the results of our present study indicate that larger diameter pins provide greater resistance to torsional stress. The diameter of the pin does make a difference in fracture stability, and although uncommonly indicated in clinical practice, the medial pin also increases fracture stability.
